# Dual Role of Interleukin-10 in Murine NZB/W F1 Lupus

**DOI:** 10.3390/ijms22031347

**Published:** 2021-01-29

**Authors:** Anaïs Amend, Natalie Wickli, Anna-Lena Schäfer, Dalina T. L. Sprenger, Rudolf A. Manz, Reinhard E. Voll, Nina Chevalier

**Affiliations:** 1 Department of Rheumatology and Clinical Immunology, Medical Centre—University of Freiburg, Faculty of Medicine, 79106 Freiburg, Germany; anais.amend@uniklinik-freiburg.de (A.A.); natalie.wickli@gmail.com (N.W.); anna-lena-schaefer@t-online.de (A.-L.S.); dalina.sprenger@uniklinik-freiburg.de (D.T.L.S.); reinhard.voll@uniklinik-freiburg.de (R.E.V.); 2 Institute for Systemic Inflammation, University of Lübeck, 23562 Lübeck, Germany; Rudolf.Manz@uksh.de

**Keywords:** IL-10, systemic lupus erythematosus, IL-10R, autoimmunity

## Abstract

As a key anti-inflammatory cytokine, IL-10 is crucial in preventing inflammatory and autoimmune diseases. However, in human and murine lupus, its role remains controversial. Our aim was to understand regulation and immunologic effects of IL-10 on different immune functions in the setting of lupus. This was explored in lupus-prone NZB/W F1 mice in vitro and vivo to understand IL-10 effects on individual immune cells as well as in the complex in vivo setting. We found pleiotropic IL-10 expression that largely increased with progressing lupus, while IL-10 receptor (IL-10R) levels remained relatively stable. In vitro experiments revealed pro- and anti-inflammatory IL-10 effects. Particularly, IL-10 decreased pro-inflammatory cytokines and slowed B cell proliferation, thereby triggering plasma cell differentiation. The frequent co-expression of ICOS, IL-21 and cMAF suggests that IL-10-producing CD4 T cells are important B cell helpers in this context. In vitro and in vivo effects of IL-10 were not fully concordant. In vivo IL-10R blockade slightly accelerated clinical lupus manifestations and immune dysregulation. Altogether, our side-by-side in vitro and in vivo comparison of the influence of IL-10 on different aspects of immunity shows that IL-10 has dual effects. Our results further reveal that the overall outcome may depend on the interplay of different factors such as target cell, inflammatory and stimulatory microenvironment, disease model and state. A comprehensive understanding of such influences is important to exploit IL-10 as a therapeutic target.

## 1. Introduction 

Interleukin (IL)-10 has emerged as a key mediator of the anti-inflammatory immune response. In the setting of infections, this is important to avoid an overwhelming immune response along with the appearance of tissue damage. In chronic inflammatory and autoimmune diseases as well as in cancerous conditions, IL-10 is involved in the regulation of the delicate balance between protective immunologic effector responses and the limitation of exaggerated inflammation as well as the maintenance of immune tolerance [1]. A failure of the latter leads to pathologic conditions, such as allergies and autoimmune diseases. Accordingly, for inflammatory bowel disease, clear beneficial effects of IL-10 are reported [2,3,4,5]. Protective, disease-mitigating influences of IL-10 through inhibition of inflammation and maintenance of self-tolerance are also reported for further immune-mediated diseases, such as psoriasis or rheumatoid arthritis, as well as allergic asthma [1,6,7,8]. In cancer, IL-10 can control tumor growth by potentiating the effects of anti-tumor CD8 T cells [9,10,11]. 

IL-10 is produced by various immune cells, including macrophages, monocytes, dendritic cells (DC), neutrophils, and CD4, CD8 T and B lymphocytes. Furthermore, IL-10 can target different cell types and exert an important regulatory role on both adaptive and innate immune responses [1,10,12]. The cellular response of IL-10 depends on its binding to the IL-10 receptor (IL-10R) and intracellular signaling cascades. The IL-10R is composed of two subunits, IL-10R1 and IL-10R2 chains [13]. IL-10R1 is mostly expressed on leukocytes and serves as a ligand binding subunit of the receptor complex. IL-10R2 is constitutively expressed in most cell types, shared by other cytokine receptors and required as an accessory chain for IL-10-induced signal transduction. IL-10R1 engagement induces its oligomerization with IL-10R2 followed by activation of JAK1 and TYK2, enabling the predominant recruitment and activation of the transcription factors STAT1, STAT3 and STAT5. In addition, other signaling cascades, such as PI3K, Akt or mTORC1, are reported to mediate IL-10 effects [14,15,16,17]. 

Due to their high IL-10R levels, monocytes and macrophages are considered the main targets. In many cases, inhibitory effects on these cells are reported. These comprise reduced pro-inflammatory cytokine production or down-regulation of co-stimulatory molecule expression [1,12,18,19,20,21]. IL-10 can also limit T cell responses, either by direct inhibitory effects or indirectly via its inhibitory function on antigen-presenting cells [22,23,24,25]. Further, IL-10 proved important in the maintenance of regulatory T cells (T_reg_) and their suppressive function [26,27]. In contrast to that, in CD8 T cells, IL-10 can increase their cytotoxic function, proliferation and interferon-γ (IFN-γ) production [28,29,30,31,32]. Such “immune-stimulatory” effects have also been reported for B cells as IL-10 could promote their survival, proliferation and differentiation [33,34,35,36].

Systemic lupus erythematosus (SLE) is a prototypical autoimmune disease of still not fully clarified etiology. Like in many other autoimmune diseases, a complex interplay of genetic and environmental factors contributes to the break of tolerance and immune dysregulation underlying disease pathology. Immune changes comprise both increased activation of autoreactive B and T cells and a dysregulated innate immune system, e.g., defective clearance of apoptotic material by phagocytes, increased IFN signature or presentation of nuclear antigen in a non-tolerogenic manner. This triggers the secretion of anti-nuclear or anti-double stranded DNA (α-dsDNA) autoantibodies that can form immune complexes inducing organ inflammation and damage, e.g., in the kidney, joints, hematological compartment or the CNS [37,38,39]. 

The role of IL-10 in SLE remains insufficiently understood, and current data are controversial both in murine and human studies [39,40,41,42,43,44,45,46,47,48]. Specifically, stimulatory IL-10 effects on B cells and autoantibody production [49,50] are suspected to be responsible for the often reported detrimental IL-10 effects on lupus pathology. Therefore, the aim of this study was to understand the regulation of IL-10 and IL-10R expression in the setting of lupus and their pro- versus anti-inflammatory effects on different target immune cells in this context. The latter was explored in vitro and in vivo to understand the potential impact of IL-10 on individual immune cell functions in comparison to the complex in vivo setting. 

## 2. Results

### 2.1. Il-10 Expression Largely Increases with Lupus Progression in NZB/W F1 Animals, While IL-10R Levels Remain Relatively Stable 

Our first aim was to understand if the expression of IL-10 and the IL-10R by various immune cell subsets changes with progressing disease. This might underlie a potentially different reactivity of individual cells towards IL-10 along with changed immune effector functions at different disease stages. 

**Figure 1:**
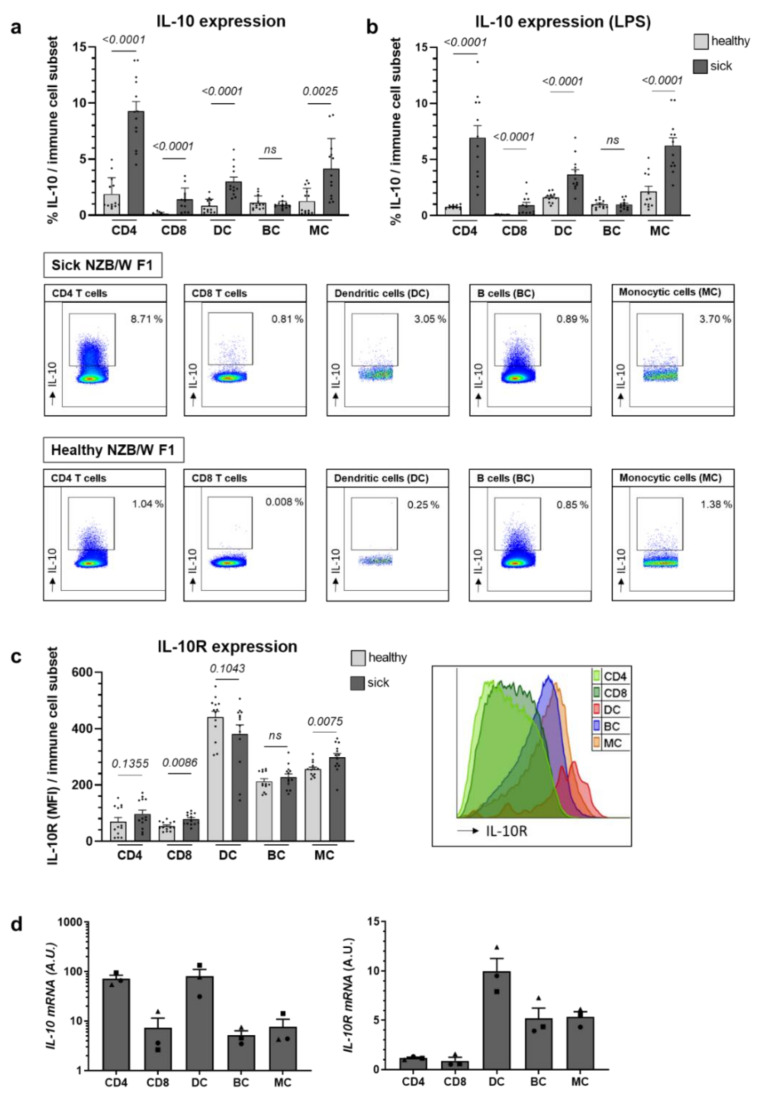
Disease-stage dependent expression of IL-10 and IL-10R on main immune cell populations in lupus-prone NZB/W F1 mice. (**a**–**c**) Splenocytes from 14 week old healthy (*n* = 12–13 mice) and 28 week old NZB/W F1 animals with established autoantibodies and beginning nephritis (sick; *n* = 12–13 mice) were (**a**) stimulated with PMA and ionomycin or (**b**) PMA, ionomycin and LPS and examined by flow cytometry for expression of IL-10 on CD4 and CD8 T cells, dendritic cells (DC), B cells (BC) and monocytic cells (MC). Representative FACS blots are shown and pooled results of three independent experiments depicted as scatter blots with mean ± SEM and with each data point representing an individual mouse. (**c**) IL-10R expression was determined on the respective cell subsets without additional stimulation. A representative histogram of the IL-10R mean fluorescence intensity (MFI) on the different subsets of 28 week old NZB/W F1 animals is shown and pooled results of three independent experiments depicted as scatter blots with mean ± SEM and with each data point representing an individual mouse. (**d**) CD4 and CD8 T cells, dendritic cells (DC), B cells (BC) and monocytic cells (MC) were sort-purified from unstimulated splenocytes of 28 week old NZB/W F1 mice to determine *mRNA* expression levels of IL-10 and IL-10R. Results are expressed as scatter blots with mean ± SEM; each data symbol represents an individual mouse. The *p* value was calculated using a Mann–Whitney test to determine the difference in the expression of IL-10 or IL-10R between healthy and diseased animals (**a**,**b**).

The expression of IL-10 was determined ex vivo by flow cytometry in main immune cell populations, CD4 and CD8 T cells, dendritic cells (DC), B cells and monocytic cells, after 4 h re-stimulation with phorbol 12-myristate-13-acetate (PMA) and ionomycin. Apart from B cells, we noted an increase in IL-10 expression in all examined immune cells in mice with established lupus (Figure 1a). As innate immune cells as well as B cells might require additional stimuli for efficient cytokine production, we included LPS-stimulation but observed no major influence on the IL-10 expression pattern (Figure 1b). Generally, under the conditions tested, CD4 T cells, followed by monocytic cells and DC, constituted the most important IL-10 producers (Figure 1a,b). As immune cell subsets may respond with differing efficiency towards the applied stimuli hampering their comparability, we also examined *IL-10 mRNA* levels from ex vivo-purified subpopulations without stimulation. This confirmed CD4 T cells as the most efficient IL-10 producers in New Zealand black × New Zealand white (NZB/W) F1 animals with established disease (Figure 1d). While monocytic cells expressed higher IL-10 levels after stimulation (Figure 1a,b), *IL-10 mRNA* transcripts were higher in unstimulated DC compared to monocytic cells (Figure 1a,b,d).

In contrast to IL-10, disease progression did not consistently increase ex vivo expression levels of IL-10R on the different immune cell subsets. IL-10R levels were examined ex vivo by flow cytometry and real-time quantitative PCR (RT-PCR), and without further stimulation. Using either method, we found the highest IL-10R expression in DC and intermediate expression levels on monocytic cells and B cells, while CD4 and CD8 T cells expressed the lowest IL-10R levels (Figure 1c,d). We also excluded major effects of the differing cell sizes of DC, monocytes and lymphocytes on IL-10R MFI levels (Supplement Figure S1). Altogether, these results show the differential expression of both IL-10 and IL-10R on main immune cell subsets. In contrast to IL-10R, the expression levels of IL-10 are generally increasing with progressing disease. 

### 2.2. In Vitro, IL-10 Predominantly Influences the Production of Pro-Inflammatory Cytokines but Has Only Sporadic Effects on Co-Stimulatory Molecule Expression in Innate Immune Cells

We next aimed to understand effects of IL-10 on the functionality of individual immune cell subsets derived from donors with manifest lupus using in vitro culture. Generally, and to best mimic physiological conditions, whole splenocytes from NZB/W F1 mice with beginning nephritis/proteinuria were cultured in the presence of different stimuli and treated with IL-10-neutralizing antibodies (α-IL-10). 

**Figure 2:**
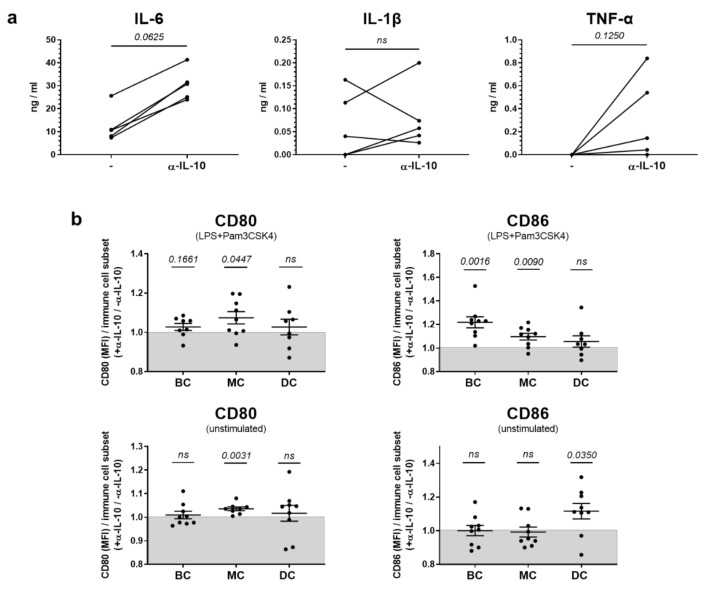
IL-10 in vitro effects on the production of pro-inflammatory cytokines and co-stimulatory molecule expression in innate immune cells. (**a**,**b**) Splenocytes from 28 week old NZB/W F1 animals with established autoantibodies and beginning nephritis were incubated for 48 h in the absence or presence of LPS and Pam3CSK4 with and without addition of α-IL-10. (**a**) Production of the inflammatory cytokines IL-6, IL-1β and TNF-α was determined by ELISA after 48 h in culture supernatants of LPS/Pam3CSK4-stimulated cells. Results are expressed as scatter plots for paired analysis. Each pair represents cells obtained from different donors (*n* = 5 mice). The *p* value was calculated using a Wilcoxon test with paired data analysis to determine the difference between α-IL-10-treated and untreated cells in individual samples. (**b**) Expression of the co-stimulatory molecules CD80 and CD86 was determined on B cells (BC), monocytic cells (MC) and dendritic cells (DC) of unstimulated and LPS/Pam3CSK4-stimulated cells. Each symbol represents results obtained from different donors (*n* = 8–9 mice) depicted as fold difference between α-IL-10- (+ α-IL-10) and untreated (− α-IL-10) cells. Results are expressed as scatter blots with mean ± SEM. The *p* value was calculated using a one-sample *t* test.

We first explored IL-10 effects on the production of inflammatory cytokines and the expression of co-stimulatory molecules that represent typical features of innate immune cells, such as DC and monocytic cells. To test IL-10 effects on the production of most important pro-inflammatory cytokines, interleukin-6 (IL-6), tumor necrosis factor α (TNF-α) and interleukin-1β (IL-1β), splenocytes from NZB/W F1 animals with beginning nephritis were stimulated for 48 h with a mix of lipopolysaccharide (LPS) and Pam3CSK4 in the presence or absence of α-IL-10. Without addition of these toll-like receptor (TLR) stimuli, no relevant cytokine levels were detectable (data not shown). In stimulated cells, addition of α-IL-10 significantly increased the production of both IL-6 and TNF-α as determined by ELISA in culture supernatants, but did not affect IL-1β levels (Figure 2a). 

In DC, monocytic cells as well as B cells, we further explored whether IL-10 affects the expression of the co-stimulatory markers CD80 and CD86 after 48 h of culture. Generally, there was a lack of consistency regarding the effects of α-IL-10 on the expression of co-stimulatory molecules. Expression levels varied under stimulatory versus non-stimulatory conditions; additionally, CD80 and CD86 expression was not uniformly influenced (Figure 2b). Only sporadically, α-IL-10 treatment increased the expression of CD80 or CD86 on the immune cell subsets examined (Figure 2b).

In sum, these results point towards rather anti-inflammatory effects of IL-10 on effector functions of innate immune cell subsets from donors with manifest lupus.

### 2.3. Among In Vitro Effects on Adaptive Immune Cells, IL-10 Most Prominently Slows B Cell Proliferation Triggering Plasma Cell Differentiation

We next tested in vitro IL-10 effects on T and B cells as representatives of adaptive immunity using the above outlined approach. In addition, in part of the experiments, we also examined the influence of recombinant TNF-α or recombinant IL-10 on B and T lymphocytes purified from splenocytes of NZB/W F1 mice to evaluate direct and indirect IL-10 effects. The rationale behind this was that our results in concert with a previous study [48] revealed inhibitory effects of IL-10 on the production of TNF-α (Figure 2a). This study hypothesized that neutralization of IL-10 protects against autoimmune development by increasing TNF-α levels [48], which is known to have diverse effects on adaptive immune cells [51,52]. 

**Figure 3:**
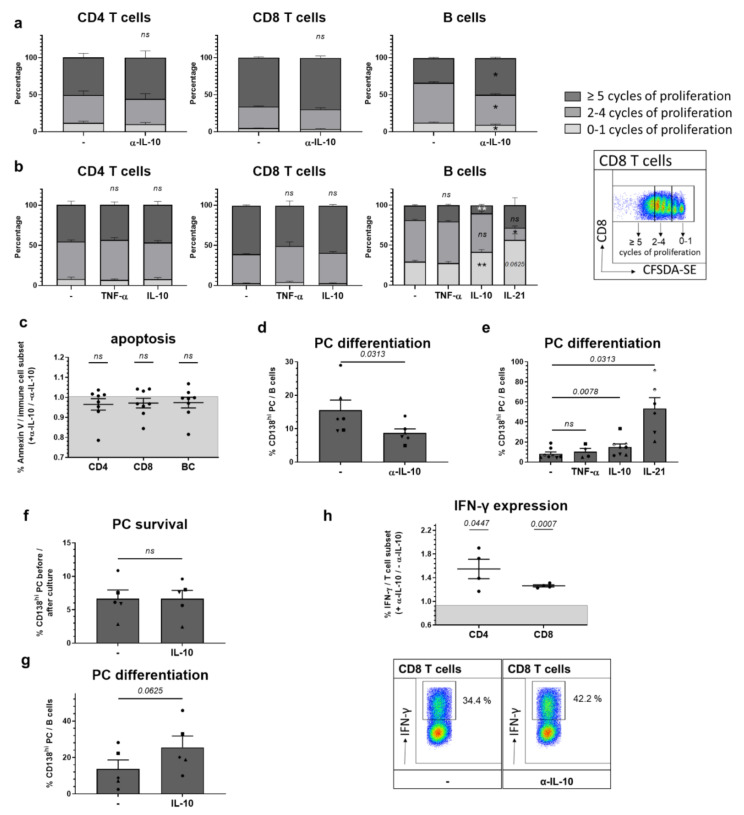
IL-10 in vitro effects on adaptive immune cells. (**a**,**d**) Splenocytes or (**b**,**e**) purified CD4, CD8 T cells or CD19^+^ B cells from 28 week old NZB/W F1 animals with established autoantibodies and beginning nephritis (*n* = 4–9 mice) were incubated for 4 days with α-CD3/CD28 (T cell stimulation) or CpG (B cell stimulation), (**a**,**d**) in the presence or absence of neutralizing α-IL-10 or (**b**,**e**) in the presence or absence of recombinant mouse IL-10, TNF-α or IL-21. (**a**,**b**) Proliferation of CD4 T, CD8 T and B cells was examined using CFDA-SE. Depicted is one representative FACS blot showing the proliferation of CD8 T cells and gating strategy to quantify cells with 0–1, 2–4 or ≥5 cycles of proliferation. The mean percentages ± SEM of cells with 0–1, 2–4 or ≥5 cycles of proliferation from 6 (**a**) and 4–9 (**b**) independent donors are expressed as stacked bars. The *p* value was calculated using a Wilcoxon test with paired data analysis to determine the difference between α-IL-10-treated and untreated cells (**a**) or the difference between untreated cells and those treated with IL-10, IL-21 or TNF-α (**b**) (* *p* < 0.05, ** *p* < 0.005). (**d**,**e**) Differentiation of B cells into CD138^hi^ plasma cells (PC) was examined by flow cytometry. The percentages of plasma cells (PC) among whole B cells (BC) are expressed as scatter blots for paired analysis; each symbol represents cells obtained from different donors. The *p* value was calculated using a Wilcoxon test with paired data analysis to determine the difference between α-IL-10-treated and untreated cells (**d**) or the difference between untreated cells and those treated with IL-10, IL-21 or TNF-α (**e**). (**c**) Splenocytes from 28 week old NZB/W F1 animals with established autoantibodies and beginning nephritis (*n* = 8 mice) were incubated for 48 h in the presence and absence of neutralizing α-IL-10, and the expression of Annexin V was determined by flow cytometry. Each symbol represents results obtained from different donors depicted as fold difference between α-IL-10- (+α-IL-10) and untreated (−α-IL-10) cells. Results are expressed as scatter blots with mean ± SEM. The *p* value was calculated using a one-sample *t* test. (**f**,**g**) CD138^hi^ PC and CD138^-^CD19^+^ B cells (*n* = 5 mice) were sort-purified and incubated for 2 and 4 days, respectively, with CpG and in the presence or absence of recombinant IL-10. (**f**) The survival of PC was determined by flow cytometry and expressed as percentage of CD138^hi^ PC after 2 days of culture/number of starts and expressed as scatter blots for paired analysis. (**g**) Differentiation of B cells into CD138^hi^ plasma cells (PC) was examined by flow cytometry, and the percentages of plasma cells (PC) among whole B cells (BC) are expressed as scatter blots for paired analysis; each data symbol represents cells obtained from different donors. The *p* value was calculated using a Wilcoxon test with paired data analysis to determine the difference between untreated cells and those treated with IL-10. (**h**) Splenocytes from 28 week old NZB/W F1 animals (*n* = 4 mice) were stimulated for 48 h with α-CD3/CD28 and in the presence or absence of neutralizing α-IL-10. Expression of IFN-γ was determined on CD4 and CD8 T cells after re-stimulation with PMA/ionomycin for an additional 4 h. Each symbol represents results obtained from different donors depicted as fold difference between α-IL-10-treated (+α-IL-10) and untreated (−α-IL-10) cells. Results are expressed as scatter blots with mean ± SEM. The *p* value was calculated using a one-sample *t* test. Depicted is also one representative FACS blot showing the expression of IFN-γ on CD8 T cells in cells treated or not with α-IL-10.

We first examined effects of IL-10 on the proliferation of CD4 and CD8 T cells, or B cells. To that end, splenocytes or purified immune cell subsets were either stimulated with α-CD3/CD28 or GPG for 3–4 days. Proliferation was measured using carboxy-fluorescein diacetate, succinimisyl ester (CFDA-SE). We noted that neutralization of IL-10 only enhanced the proliferation of B cells, while that of CD4 and CD8 T cells was not affected. In accordance with that, addition of recombinant IL-10 to cultures of purified immune cell subsets only slowed the proliferation of B cells, but did not affect T cells. Under the tested conditions, TNF-α did not influence the proliferation of B and T cells (Figure 3a,b). We also did not find that IL-10 changes the viability of B or T lymphocytes, as determined by Annexin V apoptosis staining. This was examined for splenocytes cultured for 36–48 h in the presence or absence of neutralizing α-IL-10 and without additional stimulation (Figure 3c).

While IL-10 slowed B cell proliferation, differentiation into plasma cells (PC) was enhanced (Figure 3d,e). This was consistently found in splenocyte cultures supplemented with IL-10-neutralizing antibodies as well as in purified B cells treated with recombinant IL-10. Addition of TNF-α to purified B cell cultures had no effect on plasma cell differentiation, while interleukin-21 (IL-21), a typical B-cell helper cytokine, proved more efficient than IL-10 in inducing a differentiation of plasma cells from B cells and in slowing B cell proliferation (Figure 3b,d,e). 

As splenocyte cultures and purified CD19^+^ cells contain both B cells and differentiated plasma cells, we aimed to determine if the triggering effects of IL-10 on plasma cell frequencies in the respective cultures resulted from improved survival of preformed plasma cells or a plasma cell differentiation from B cells. Therefore, we purified CD138^hi^ PC and CD138^−^CD19^+^ B cells that were cultured for 2 or 4 days, respectively, in the presence of CpG with and without addition of recombinant IL-10. Already after 2 days in culture, the majority of cultured plasma cells had died, irrespective of the presence of IL-10 (Figure 3f). Vice versa, IL-10 increased the differentiation of CD138^−^CD19^+^ B cells into plasma cells (Figure 3g). 

We further tested the influence of IL-10 on phenotypic changes of CD4 and CD8 T cells. After 2 days of culture in the presence of α-CD3/CD28 with and without α-IL-10, we examined their expression of IFN-γ. We found that neutralizing IL-10 increased the expression of IFN-γ in both CD4 and CD8 T cells (Figure 3h). 

To summarize, most pronounced among the in vitro effects of IL-10 on adaptive immune cells was the reduction in B cell proliferation and the concomitant increase in plasma cell differentiation. For innate immune cells, rather anti-inflammatory effects of IL-10 were found. A higher responsiveness of these cell subsets compared to T cells might be due to their relatively higher expression of the IL-10R. However, an impact of the different stimulatory conditions also needs to be considered. Altogether, these data show that pro- and anti-inflammatory IL-10 effects occur simultaneously.

### 2.4. High Expression of IL-10 Is Found in ICOS^+^ and PD1^+^ Effector CD4 T Cells and Co-Incites with Increased Levels of IL-21, cMAF and IFN-γ

**Figure 4:**
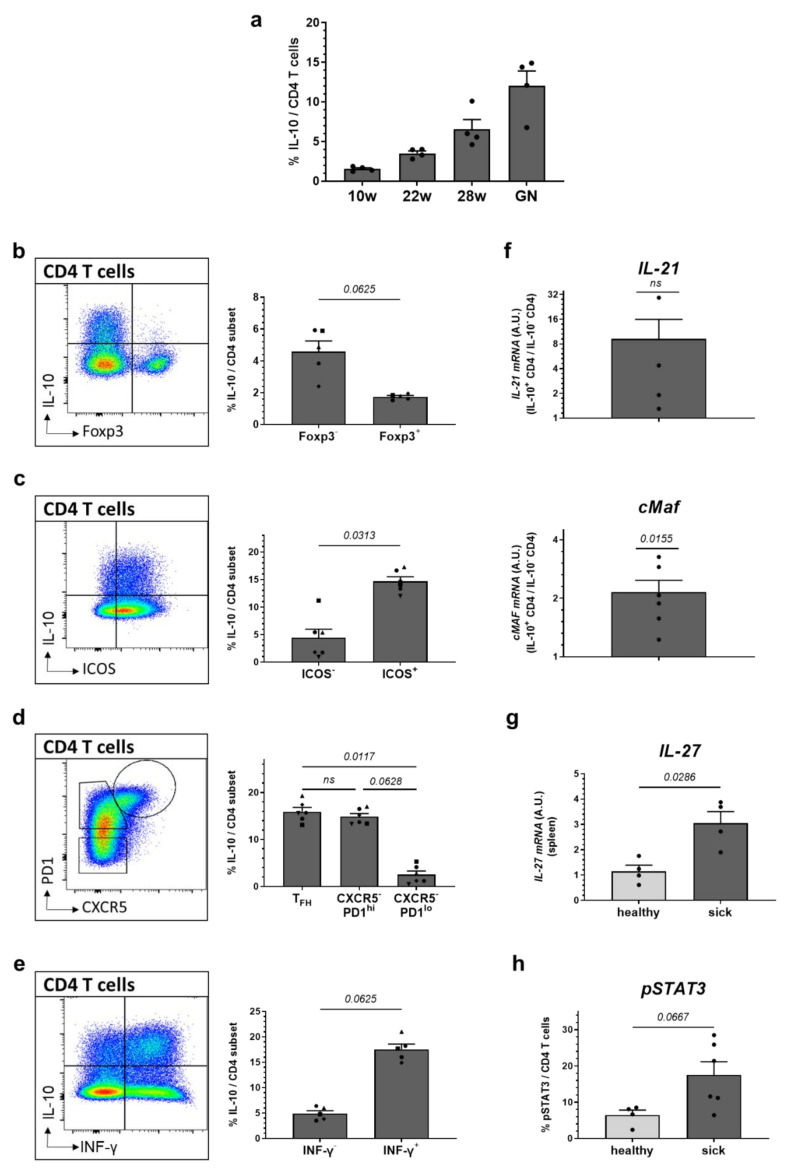
Characterization of IL-10-expressing CD4 T cells. (**a**) Splenocytes from NZB/W F1 animals (*n* = 4 mice) were examined at the age of 10 weeks, 22 weeks, 28 weeks and with established glomerulonephritis (GN) for IL-10 expression in CD4 T cells by flow cytometry. (**b**–**e**) Expression of IL-10 was examined in (**b**) FoxP3^+^ T_reg_ and FoxP3^−^ effector CD4 T cells; (**c**) ICOS^+^ versus ICOS^−^ CD4 T cells; (**d**) CXCR5^hi^PD1^hi^ T_FH_, CXCR5^−^PD1^hi^ and CXCR5^−^PD1^lo^ CD4 T cells; and (**e**) in relation to IFN-γ expression in CD4 T cells from splenocytes of 28 week old NZB/W F1 animals with established autoantibodies and beginning nephritis by flow cytometry (*n* = 5–6 mice). Results are expressed as scatter blots with mean ± SEM, and representative FACS blots are depicted; each data point (**a**) or data symbol (**b**–**e**) represents an individual mouse. The *p* value was calculated using a Wilcoxon test (**b**,**c**,**e**) or Friedman test for paired analysis (**d**). (**f**) *IL-21* and *cMAF mRNA* expression was determined in IL-10^+^ and IL-10^-^ CD4 T cells after purification from splenocytes of 28 week old NZB/W F1 animals with established autoantibodies and beginning nephritis (*n* = 4–6 mice). Each symbol represents results obtained from different donors depicted as fold difference between IL-10^+^ and IL-10^−^ CD4 T cells. Results are expressed as scatter blots with mean ± SEM. The *p* value was calculated using a one-sample *t* test. (**g**) *IL-27 mRNA* expression was examined in spleens of healthy, 10 week old (*n* = 4 mice) and sick, 28 week old (*n* = 4 mice) NZB/W F1 animals. (**h**) Expression of pSTAT3 on CD4 T cells was determined in splenocytes of healthy, 10 week old (*n* = 4 mice) and sick, 28 week old (sick; *n* = 6 mice) NZB/WF1 animals by flow cytometry. (**g**,**h**) Results are expressed as scatter blots with mean ± SEM; each data point represents an individual mouse. The *p* value was calculated using a Mann–Whitney test.

In view of the fact that IL-10 propagated the in vitro differentiation of B cells into plasma cells and, as CD4 T cells are important B cell helpers and show pronounced up-regulation of IL-10 with progressing disease (Figure 4a), we examined whether IL-10 expression in CD4 T cells from donors with manifest lupus coincides with further B cell-helper molecules.

By co-expression analysis using flow cytometry, we found that FoxP3^−^ effector rather than FoxP3^+^ regulatory T cells (T_reg_) represent the main IL-10-producers (Figure 4b). We further examined IL-10 expression in follicular B-helper T cells (T_FH_) that are important for efficient germinal center (GC) formation and involved in the production of class-switched, high affinity immunoglobulin G (IgG) autoantibodies [53]. We found high IL-10 co-expression in PD1^+^ and ICOS^+^ CD4 T cells; however, no specific accumulation of IL-10-expressing CD4 T cells in bona-fide CXCR5^hi^PD1^hi^ T_FH_ cells compared to CXCR5^−^PD1^hi^ CD4 T cells was found (Figure 4c,d). As a further B cell help-associated feature, we explored the expression of *IL-21* via RT-PCR in IL-10-expressing CD4 T cells after re-stimulation with PMA and ionomycin and fluorescence-activated cell sorting (FACS)-purification using an IL-10-capture assay. Interestingly, we noted an increased co-expression of *IL-21* as well as of the transcription factor *cMAF* in IL-10-producing CD4 T cells compared to their IL-10^−^ counterparts (Figure 4f). In addition to that, a strong co-expression of IFN-γ and IL-10 was noted (Figure 4e). To conclude, the high co-expression of ICOS, *IL-21* and IFN-γ in IL-10^+^ CD4 T cells might equip these cells with increased B cell helper qualities. We further explored a possible connection between interleukin-27 (IL-27), STAT3 signaling and IL-10 expression in CD4 T cells of mice with manifest lupus and found that *IL-27* transcripts were increased in the spleens of ill NZB/W F1 mice compared to healthy animals (Figure 4g). Likewise, NZB/W F1 mice with established lupus show higher pSTAT3 expression in CD4 T cells (Figure 4h).

### 2.5. In Vivo Administration of α-IL-10R to NZB/W F1 Animals with Beginning Lupus Goes along with Moderate Immunologic Changes and Slightly Accelerates Disease Progression

The above in vitro approach allowed us to explore individual IL-10 effects on main immune cell subsets from donors with manifest lupus and showed the co-existence of pro- and anti-inflammatory effects. To explore IL-10 effects on immune cells and disease in the inflamed in vivo setting of lupus, 20–22 week old NZB/W F1 animals with detectable autoantibody titers were treated with anti-IL-10R antibodies (α-IL-10R) or isotype control at a dose of 500 µg every 3 weeks over 6 weeks. 

**Figure 5:**
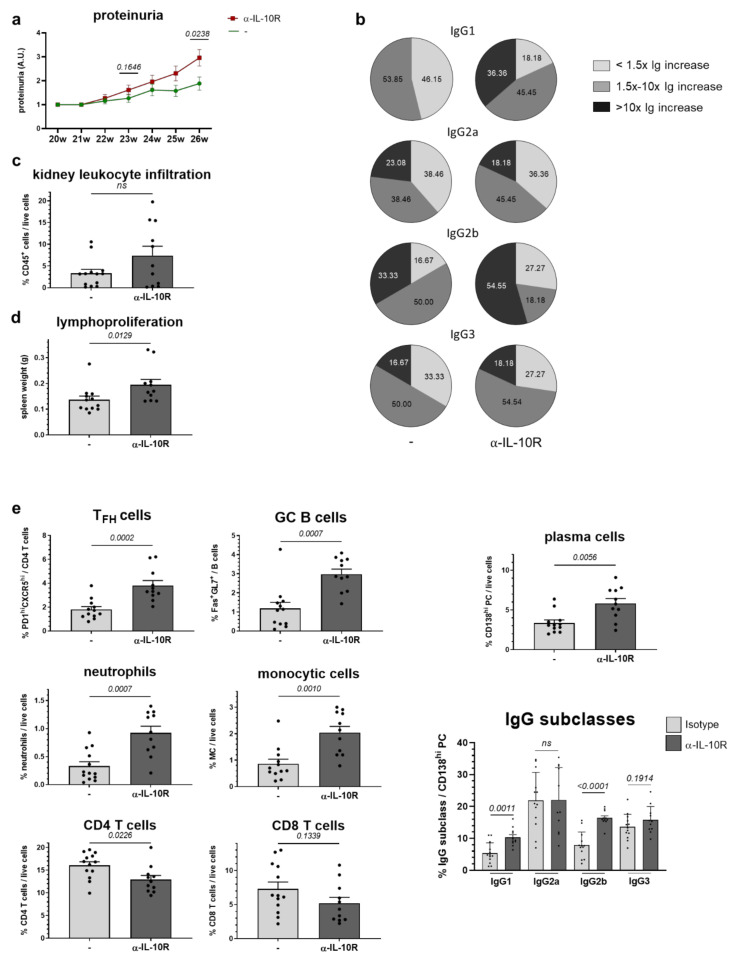
In vivo effects of α-IL-10R application on lupus pathology and immune status in NZB/W F1 animals. 20–22 week old NZB/W F1 animals with detectable α-dsDNA antibodies were treated with α-IL-10R or isotype control (*n* (α-IL-10R) = 11–13 mice; *n* (isotype) = 13 mice) at a dose of 500 µg every 3 weeks over 6 weeks. Clinical disease progression and impact on immunologic changes were determined by: (**a**) development of proteinuria depicted as trend line with mean ± SEM for each time point; (**b**) levels of α-dsDNA IgG1, IgG2a, IgG2b and IgG3 before and after treatment; indicated are the percentages of animals with an α-dsDNA titer increase >10×, of 1,5–10× and <1,5× as pie graph; (**c**) kidney infiltration by CD45^+^ leukocytes; (**d**) lymphoproliferation by splenomegaly; and (**e**) immune status evaluation using flow cytometry in spleen 1 week after termination of α-IL-10R or isotype treatment; depicted are relative changes in neutrophils, monocytic cells and CD4 and CD8 T cells, in CXCR5^hi^PD1^hi^ T_FH_, Fas^hi^GL7^hi^ GC B cells, CD138^hi^ plasma cells and their expression of IgG1, IgG2a, IgG2b and IgG3. (**a**,**c**–**e**) The *p* value was calculated using a Mann–Whitney test to determine the difference between isotype- and α-IL-10R-treated animals. Results are expressed as scatter blots with mean ± SEM; each data point represents an individual mouse.

First, we noted accelerated proteinuria development under α-IL-10R treatment (Figure 5a). Additionally, we examined α-dsDNA autoantibody titers pre- and post-treatment (Figure 5b). Treatment with α-IL-10R increased α-dsDNA-IgG1 and -IgG2b production, but had no effect on α-dsDNA-IgG2a and -IgG3 serum levels. These mildly disease-triggering effects of α-IL-10R treatment are also reflected in a slightly higher kidney infiltration by CD45^+^ leukocytes and splenomegaly indicative of lymphoproliferation (Figure 5d).

We next explored how closely the clinical influence of IL-10R-blockade correlates with changes of innate and adaptive immunity. As reported in detail elsewhere [54], we confirmed in this study that progressing disease goes along with significant immunologic changes in NZB/W F1 mice comprising a relative increase in neutrophils and DC and a relative decrease in CD4 and CD8 T cells (Table 1). Generally, in animals with established disease, T cells show elevated expression levels of IFN-γ and the activation marker CD44; CD4 T cells also display higher proportions of CXCR5^hi^PD1 T_FH_ cells and an increased T_reg_ differentiation and IL-10 expression. The B cell compartment was marked by enhanced GC B cell and plasma cell differentiation as well as higher IgG expression (Table 1). 

Consistent with the mild disease-triggering effects, in α-IL-10R-treated mice, we noted a greater appearance of immunologic changes present in sick compared to healthy mice (Table 1). Generally, the immunologic changes may result from advanced disease in these mice, alternatively from direct effects of the α-IL-10R treatment. Under the here employed experimental conditions, a clear distinction between the two scenarios is not possible. Compared to matched, isotype control-treated animals, we found a relative increase in neutrophils and monocytic cells in the spleens of α-IL-10R-treated animals (Table 1, Figure 5e). In accord with our in vitro data, we did not find clear changes in the expression of the co-stimulatory molecules CD80 and CD86 as well as I-A/I-E or ICOSL in DC, monocytic cells or B cells (Table 1). A relative increase in neutrophils was also found in NZB/W F1 animals with advanced disease compared to young, yet healthy mice (Table 1). As discussed above, this might reflect the slightly more advanced disease in mice treated with α-IL-10R antibodies; alternatively, IL-10 was reported to inhibit neutrophil migration [55]. A relative decline in CD4 T cells, and in CD8 T cells to a lesser extent, further support this assumption. In contrast, we did not find an increase in the expression of inflammatory IFN-γ or the activation marker CD44, nor an elevation in IL-10 expression or T_reg_ frequencies, as found in diseased compared to healthy mice (Table 1, Figure 5e). As observed in vitro, α-IL-10R application enhanced B cell proliferation; in contrast to this, it increased plasma cell numbers and their IgG2b expression. Along with the pronounced increase in T_FH_ and GC B cells, this supports the disease-triggering effects of α-IL-10R treatment (Table 1, Figure 5e). In contrast to our in vitro data, in vivo IL-10R blockade increased not only B cell but also CD4 and CD8 T cell proliferation as measured by bromdesoxyuridin (BrdU) incorporation. The observed slight decline in T cells could be explained by an increased susceptibility to apoptosis, as we found higher Annexin V-binding in CD4 and CD8 T cells of α-IL-10R-treated mice (Table 1), which contrasts with our in vitro results (Figure 3c). 

To summarize, our results suggest that a blockade of the IL-10R in vivo slightly accelerates lupus progression when applied to animals with established autoantibodies. This is reflected in a higher level of immune dysregulation that might either be related to advanced disease or direct α-IL-10R effects. Generally, we observed contrasting effects of IL-10-antagonism in vitro and in vivo. Altogether, these observations suggest diverse pro- and anti-inflammatory immune modulatory effects of IL-10 that may vary depending on the inflammatory micro-environment in vivo as well as stimulatory in vitro conditions. Additionally, in the complex in vivo setting, some immunologic effects might be covered or overruled by other, more stringent effects. 

## 3. Discussion

The central question of this study was to explore the regulation of IL-10 and IL-10R expression and IL-10 effects on main immune cells and disease pathology in the setting of lupus. We found that disease progression correlated with an up-regulation of IL-10 production in most immune cells, but only marginally influenced their IL-10R expression levels. In vitro, we noted both inhibitory and immune-triggering IL-10 effects. While IL-10 reduced the production of pro-inflammatory cytokines, it slowed B cell proliferation, thereby triggering plasma cell differentiation. We assume that IL-10-expressing CD4 T cells might play a role in this regard, as their frequencies increase with progressing disease and show a prominent co-expression of B cell helper molecules such as ICOS, *IL-21*, *cMAF* and IFN-γ. However, in vivo IL-10R blockade in mice with beginning lupus slightly accelerated disease progression, suggesting a rather protective role of IL-10 under these circumstances. Clinical effects were also reflected in immunologic changes. The fact that these changes partly differed from the observed in vitro effects of IL-10 might indicate that the diverse immune-modulatory effects of IL-10 are influenced by the inflammatory microenvironment or varying stimulatory conditions. Accordingly, in the complex in vivo setting, IL-10-related B-helper qualities as found in vitro might potentially be overruled by prevailing anti-inflammatory IL-10 effects on other immune cells, e.g., on T_FH_ or T_reg_ cells.

The increasing expression of IL-10 in NZB/W F1 mice with progressing disease indicates a potential impact of IL-10 on lupus pathogenesis and is in concert with other studies. These show IL-10 overproduction in sera and immune cells from SLE patients and a correlation with disease activity [41,42,56,57,58,59,60,61,62]. In concert with studies using B6.TC lupus mice [63], we identified CD4 T cells as an important source of IL-10. In contrast, other studies report a predominant IL-10 production by monocytes and B cells [64]. We assume that increased production of *IL-27* and elevated pSTAT3 levels underlie increased IL-10 expression in CD4 T cells of ill compared to healthy NZB/W F1 mice [65], rather than genetic [59,66,67,68] or epigenetic [69] factors. 

Given the plasma cell-inducing effects of IL-10 in vitro, it is possible that IL-10^+^ CD4 T cells mediate important B-helper functions. In accordance with our studies, among IL-10^+^ CD4 T cells accumulating in lupus-prone mice and SLE patients [70,71,72], phenotypes distinct from T_reg_ and/or CXCR5^hi^PD1^hi^ T_FH_ were noted [70,73]. Specifically, an IFN-γ^+^CXCR5^−^CXCR3^+^PD1^hi^ CD4 T cell population was expanded in the blood and kidneys of SLE patients and found to provide B cell help via IL-10 [73]. Considering these studies and the here observed strong co-expression of IFN-γ, IL-21, ICOS and cMAF in IL-10^+^ CD4 T cells, one might speculate that a proportion of IL-10^+^ CD4 T cells may represent a specialized B-helper T cell subset. However, the co-expression of PD1, ICOS and c-MAF was also reported for FoxP3^−^ inducible T regulatory type 1 (TR1) cells. Furthermore, IL-27 and pSTAT3 signaling, found to be increased in our study, have been suggested to be involved in TR1 cell differentiation [39,74]. In the context of autoimmunity, TR1 cells might importantly contribute to immune tolerance, and in contrast to IL-10^+^ B-helper T cells, mediate disease control.

In contrast to the consistent increase in IL-10 in human and murine lupus, its impact on disease pathology is discussed controversially. While continuous administration of α-IL-10 from 4 weeks of age onwards could delay autoantibody formation and lupus manifestation in NZB/W F1 animals [48], a deficiency of IL-10 in MRL-Fas^lpr^ mice induced lupus symptoms and autoantibodies [47]. The same beneficial IL-10 effects were reported for B6.TC lupus mice [63]. In humans, in vivo effects of therapeutic IL-10-neutralization have only been tested on a cohort of six lupus patients, leading to a reduction in disease severity [45]. Vice versa, α-IL-10 antibodies were found in sera of SLE patients and were related to high levels of serum IgG, but not associated with disease activity [43]. These contradictory results point out that effects of IL-10 may vary depending on the applied disease model. In addition, our study suggests that timing of treatment and disease stage may dictate the outcome of an IL-10-neutralizing therapy. In contrast to the continuous neutralization of IL-10 in the study of Ishida et al. [48], we chose to treat mice with beginning disease as that might more closely reflect the situation in clinical practice. Additionally, a different neutralizing antibody was applied in our study and only for a treatment period of 6 weeks to explore immune status in relation to clinical disease in our study. Although not reaching overall statistical significance, our data indicate rather protective effects of IL-10 when applied at later disease stages. In accordance with our study, the application of in vitro-generated polyclonal IL-10-producing CD4 T cells to severely-ill NZB/W F1 mice improved lupus pathology [75]. 

An important question is what mechanistically links IL-10 to these differing outcomes. IL-10R expression critically influences the responsiveness of cells towards IL-10 [76]; its relevance in human and murine lupus remains, however, controversial [77,78,79]. A largely equal IL-10R expression in main immune cells in ill compared to healthy NZB/W F1 mice argues against the assumption that skewed IL-10R levels may underlie differing, e.g., disease stage-dependent, effects of IL-10. Alternatively, the influence of external factors—e.g., disease model, individual patient, anatomical site, inflammatory microenvironment, stimulatory condition and disease stage—might dictate the overall outcome of IL-10 effects on immunologic changes and shift disease in one direction or another. 

TNF-α could be an important player in this context and like IL-10 exert context-dependent beneficial or detrimental effects. In NZB/W F1 mice, the delayed onset of autoimmunity in the presence of neutralizing α-IL-10 was suspected to be due to an up-regulation of endogenous TNF-α [48]. In NZB/W F1 mice, TNF-α exerts a protective role when replaced at low doses [80,81], possibly due to decreasing major histocompatibility complex (MHC) class II expression [82,83]. Although IL-10-neutralization also reduced TNF-α in our study, we noted neither a clear effect on MHC class II expression nor on other co-stimulatory molecules. We also found no TNF-α effect on plasma cell differentiation or lymphocyte proliferation upon supplementation in the employed in vitro cultures. This is in contrast to studies reporting that TNF-α can activate T cells and the production of autoantibodies [84] and even selectively destroy autoreactive T cells in several autoimmune diseases [85,86,87,88,89]. The dependence of TNF-α and IL-10 effects on disease model and stage is further illustrated by the following examples: In contrast to NZB/W F1 mice [80,81], TNF-α was overexpressed in the serum and kidneys of MRL/lpr mice and correlated with inflammatory organ disease. Moreover, TNF-α treatment deteriorated lupus manifestation and progression in MRL/lpr and BXSB strains [80,82,90,91]. Interestingly, low doses of TNF-α, when administered exclusively at late disease stages to NZB/W F1 mice, accelerated renal disease [92,93]. Given that IL-10 suppresses TNF-α, this might, in part, explain the rather disease-triggering effects observed in α-IL-10R-treated NZB/W F1 animals with beginning lupus in our study compared to the contrasting effects of α-IL-10 treatment started at an early age reported by Ishida et al. [48].

Apart from disease model and stage, in vitro stimulatory and in vivo micro-environmental conditions may dictate the dual role of IL-10 and yield contrasting immunologic effects. Accordingly, there is some inconsistency regarding the effects of IL-10 on T cell activation or IFN-γ expression. Continuous IL-10 overexpression in B6.TC mice significantly reduced T cell activation [63]. In lupus-prone MRL/lpr animals, IL-10 exerted protective effects by suppressing pathogenic T_h1_ responses and IFN-γ production [47]. In contrast to that, in the setting of cancerous disease and depending on the stimulatory conditions, IL-10 could or could not induce CD8 T cell activation [94,95]. In our study, in vitro neutralization of IL-10 reduced IFN-γ expression in T cells, while in vivo administration of α-IL-10R had no impact. A further example of the impact of in vitro stimulatory conditions in our study is the inconsistent effects of α-IL-10 on CD80 and CD86 expression when splenocytes were incubated with and without additional TLR1/2/4 stimulation. 

While the in vitro approach allows us to dissect specific effects on individual cell subsets, due to its multifaceted role, it is probable that in the complex in vivo setting, various IL-10 effects occur simultaneously. Some of those might not be detectable as they are covered or overruled by other, more stringent effects. In our study, this scenario could apply to B cells and their differentiation into plasma cells. Both in vitro and in vivo, IL-10 slowed B cell proliferation, but only under the tested in vitro conditions it also increased plasma cell differentiation. In vivo, the opposite scenario was observed, as IL-10R neutralization increased plasma cell differentiation and their IgG1 and IgG2b expression. Based on the collected data, we could not definitely clarify the key immunologic mechanisms underlying these beneficial anti-inflammatory in vivo IL-10 effects in our model. In part, these may also merge with immunologic effects occurring with progressing disease. We suspect predominant anti-inflammatory effects of IL-10 on the T cell compartment, especially on T_reg_ and T_FH_ cells. Our assumptions are based on the finding that T_reg_ frequencies increase with progressing disease. However, despite the fact that α-IL-10R- compared to isotype-treated animals showed advanced disease, their T_reg_ frequencies were lower, possibly going along with reduced suppressive effects on expanded T_FH_ cells and GC B cells. In addition to that, IL-10 can exert direct inhibitory effects on T_FH_ cells. For instance, in patients with Sjögren’s syndrome, T_FH_ cells are critically restrained by IL-10-producing regulatory B cells (B_reg_) [96]. IL-10-producing B_reg_ could also maintain T_reg_ function and control T_h1_/T_h17_ effector responses [97,98,99]. 

At first sight, the observation that in vivo α-IL-10R treatment slightly triggered lupus progression without increasing IL-10 and IFN-γ expression in T cells was paradoxical. This would suggest stimulatory IL-10 effects on IFN-γ expression as well as the expression of IL-10 itself. Consistent with this idea, previous studies have shown that IL-10R signaling can facilitate its own production via STAT3 phosphorylation [26] indicative of a feed-forward IL-10-loop. Likewise, IL-10-incompetent CD4 T cells failed to secrete IFN-γ and were inefficient in T_h1_ commitment [100]. The mechanism of T_h1_ cell autoregulation through co-induction of IL-10 and IFN-γ might be a further explanation for the observed strong co-expression of IL-10 and IFN-γ in mice with lupus manifestation. This transition of an ”IFN-γ only into an IFN-γ—IL-10 co-expression T_h1_ state” might represent an important intrinsic self-regulatory mechanism of T_h1_ cells to shut down their effector functions, limiting overwhelming inflammation and autoimmunity [101]. Which downstream signaling pathways mediate such stimulatory versus suppressive pathways and to what extent this is impacted by the inflamed setting of active lupus or other diseases remains unresolved but represents an interesting putative target to manipulate inflammatory diseases.

To conclude, IL-10 has emerged as a major suppressor of the immune response and a key player in human disease. However, there is evidence that IL-10 may play a previously underappreciated dual role, in some contexts stimulating the immune response and yielding disease-triggering effects instead of suppressing it. This may depend on factors such as the cell types targeted and differing contexts such as micro-environmental inflammation, anatomical location, disease type, manifestation and stage. In view of these differing effects, the pre-clinical success of IL-10 has been conflicting. However, a deeper understanding of the pleiotropic nature of this cytokine and fine-tuning of several variables might enable its consideration for clinical application. Hence, more research is needed to elucidate dual IL-10 effects. Detailed insight into this may, for example, allow the use of IL-10 in combinatory therapies that selectively exploit its beneficial effects while deactivating its adverse effects.

## 4. Materials and Methods 

### 4.1. Mice and Models

Animal experiments were approved by the local governmental commission for animal protection of Freiburg (Regierungspräsidium Freiburg, approval no. G19/21, G16-58). Lupus-prone NZB/W F1 mice were generated by crossing *NZB*/BlNJ with *NZW/LacJ* mice. These were purchased from The Jackson Laboratory. For all experiments, female mice were housed on a 12 h light/dark cycle, with food and water ad libitum. The in vivo influence of IL-10 was tested by i.p. application of α-IL-10R at a dose of 500 µg every 3 weeks over 6 weeks (2 injections). Blood and urine were collected and mice euthanized at defined time points for organ harvest and downstream experiments. Mice were regularly monitored and euthanized when reaching defined ethical endpoints (proteinuria plus deteriorating general health condition and/or significant weight loss). 

### 4.2. Assessment of Proteinuria

Urine samples were collected by spontaneous urination. For a semi-quantitative measurement of proteinuria, Albustix test strips (Siemens Healthcare Diagnostics Products, Schwalbach am Taunus, Germany) were used. According to the color scale provided by the manufacturer, albuminuria was categorized as follows: 0–1 = trace, 1 = 30, 2 = 100, 3 = 300 and 4 > 2000 mg/dL. 

### 4.3. Assessment of α-dsDNA Autoantibodies and IgG Subclasses (ELISA)

IgG1, IgG2a, IgG2b and IgG3 antibody secretion directed against dsDNA was determined by enzyme-linked immunosorbent assay (ELISA). Briefly, 384-well microtiter plates (Greiner Bio On, Frickenhausen, Germany) were pre-coated with 20 µg/mL Poly-L-Lysin (Sigma-Aldrich, Chemie, Taufkirchen, Germany) for 1 h at 37 °C followed by coating with 20 µg/mL calf thymus DNA (Sigma-Aldrich) at 4 °C o.n. Plates were blocked with 2% fetal calf serum (FCS) in PBS for 2 h at RT. Samples were diluted in 2% FCS in PBS and incubated for 2 h at RT. Bound α-dsDNA immunoglobulins were detected with HRP-conjugated secondary antibodies specific for mouse IgG1, IgG2a, IgG2b or IgG3 (Southern Biotech, Birmingham, Alabama, USA), followed by development with TMB substrate (Thermo Fisher Scientific, Freiburg im Breisgau, Germany) according to the manufacturer’s protocol. The absorbance at 450 nm was measured using the Spark® 10 M multimode microplate reader (Tecan, Crailsheim, Germany). To determine autoantibody titers, expressed as arbitrary units (A.U.), reference sera were used to create a standard curve.

### 4.4. Cell Purification 

Mice were euthanized by CO_2_ inhalation, and after perfusion with sterile PBS, organs were collected for further analysis. Single cell suspensions of spleen and kidney were obtained by mechanic dissociation followed by red blood cell lysis. B cells, CD4 and CD8 T cells were isolated by labelling splenocytes with Biotin-conjugated αCD4, αCD8 and αCD19 antibodies in a 1st step and Biotin-specific microbeads (Miltenyi Biotec, Bergisch Gladbach, Germany) in a 2nd step. Automated positive cell isolation was performed with the autoMACS^®^ Pro Separator. Alternatively, splenocytes were labelled with Fluorochrome-labelled antibodies and sorted with a FACSAria (BD Biosciences, Heidelberg, Germany) or MoFlo Asterios Sorter as CD138^−^CD19^+^ Bells, CD138^hi^ plasma cells, B220^+^TCRβ^−^CD4^−^CD8^−^CD11b^−^NK1.1^−^ B cells, B220^−^TCRβ^+^CD4^+^CD8^−^CD11b^−^NK1.1^−^ CD4 T cells, B220^−^TCRβ^+^CD4^−^CD8^+^CD11b^−^NK1.1^−^ CD8 T cells, TCRβ^−^B220^−^NK1.1.^−^CD11b^+^CD11c^−^ monocytic cells and TCRβ^−^B220^−^NK1.1^−^CD11b^+^CD11c^hi^ dendritic cells (DC). To detect and isolate IL-10-secreting CD4 T cells, splenocytes were stimulated for 4 h with PMA (50 ng/mL) and ionomycin (1 µg/mL; both Sigma-Aldrich) and then labelled according to the manufacturer’s instructions (Miltenyi Biotec Mouse IL-10 Secretion Assay Detection Kit), followed by surface staining to detect TCRβ and CD4. TCRβ^+^CD4^+^IL-10^+^ and IL-10^−^ cells were then FACS-purified. 

### 4.5. Cell Culture 

Cells (splenocytes or purified immune cells) were cultured in RPMI 1640 medium supplemented with 2 mM glutamine, 1% (*v*/*v*) nonessential amino acids, 1% (*v*/*v*) sodium pyruvate, penicillin (50 U/mL), streptomycin (50 μg/mL) (all from Life Technologies, Thermo Fisher Scientific, Freiburg im Breisgau, Germany), 10% (*v*/*v*) FBS (anprotec, AC-SM-0161, Bruckberg, Germany) and 55 μM 2ME (Sigma-Aldrich). To determine the influence of IL-10, 10 µg/mL neutralizing α-IL-10 antibodies (α-IL-10) (BioLegend, Koblenz, Germany, clone JES5-2A5) were added to splenocyte cultures or 10 ng/mL recombinant murine IL-10 (BioLegend), IL-21 or TNF-α (PeproTech, Hamburg, Deutschland) to cultures with purified cells. To examine in vitro proliferation, cultured cells were stained with CFDA-SE (Thermofisher). To analyze proliferation of B and T cells, as well as plasma cell differentiation of B cells, splenocytes or purified cells were cultured in the presence of 1 µg/mL CpG (ODN2006, InvivoGen, Toulouse, France) or plate-bound α-CD3/CD28 (2 µg/mL, BioLegend) for 4 days in the presence or absence of neutralizing α-IL-10. To explore the expression of Annexin V, cells were cultured for 48 h without further stimulation. The expression of co-stimulatory molecules in innate immune cell subsets or cytokine secretion in splenocyte cultures was determined in the presence or absence of LPS (100 ng/mL, *E. coli* O55:B5, Sigma-Aldrich) and Pam3CSK4 (100 ng/mL, InvivoGen, tlrl-pms). The expression of IFN-γ on CD4 and CD8 T cells was examined after 2 days stimulation using plate-bound α-CD3/CD28 (2 µg/mL, BioLegend) in the presence or absence of neutralizing α-IL-10, followed by re-stimulation with PMA/ionomycin/Brefeldin A for intracellular cytokine detection as outlined below. 

### 4.6. Flow Cytometry 

Single cell suspensions of spleen and kidney were obtained by mechanic dissociation. Following incubation with α-CD16/32 antibodies (101330, BioLegend, Koblenz, Germany) to block non-specific Fc receptor binding, single cell suspensions or purified cells were stained with biotin- or fluorochrome-conjugated monoclonal antibodies diluted in 2% FCS/PBS for 30 min on ice. For intracellular or intranuclear staining, cells were fixed and permeabilized with BD Cytofix/Cytoperm (BD Biosciences, Heidelberg, Germany, 554722) or eBioscience FoxP3/Transcription Factor Staining Buffer Set (Thermo Fisher, 00552300), respectively. For intracellular cytokine staining, cells were re-stimulated with 50 ng/mL PMA (Sigma-Aldrich), 1 µg/mL ionomycin (Sigma-Aldrich) and Brefeldin A (BFA) (eBioscience, Thermo Fisher Scientific, Freiburg im Breisgau, Germany) in the presence and absence of LPS (2.5 µg/mL, *E. coli* O55:B5, Sigma-Aldrich) for 4 h at 37 °C/5%CO_2_ prior to staining and fixation. To identify apoptotic cells, Annexin V staining was performed using Annexin V Binding Buffer (BD Biosciences). In vivo proliferation was determined using a BrdU Flow Kit (BD Biosciences, Heidelberg, Germany) 48 h after injecting mice with 100 µL of 10 mg/mL BrdU/10 g body weight. Detection of pSTAT3 was performed after surface staining (as outlined above) and sequential fixation steps, including a 10 min incubation at 37 °C in BD Cytofix fixation buffer (554655), followed by a 30 min incubation on ice using BD Phosphoflow Perm III (558050). Prior pSTAT3 staining cells were rested o.n. at 37 °C. 

### 4.7. Antibodies Used for Flow Cytometry 

The following antibodies were used for flow cytometric analysis and cell sorting: TCRβ chain Biotin (109203, BioLegend, Koblenz, Germany), CD19 Biotin (115505, BioLegend, Koblenz, Germany), NK1.1 Biotin (108704, BioLegend, Koblenz, Germany), Ly6G Biotin (127604, BioLegend, Koblenz, Germany), CXCR5 Biotin (551960, BD Biosciences, Heidelberg, Germany), κ light chain Biotin (559750, BD Biosciences, Heidelberg, Germany), λ light chain Biotin (553433, BD Biosciences, Heidelberg, Germany), CD11c APC (170111482, eBioscience, Thermo Fisher Scientific, Freiburg im Breisgau, Germany), CD11b FITC (101205, BioLegend, Koblenz, Germany), Ly6G V450 (560603, BD Biosciences), Ly6C PE-Cy7 (560593, BD Biosciences), CD45 APC-Cy7 (103115, BioLegend), Streptavidin-PerCP-Cy5.5 (45431782, BD Biosciences), CD45R/B220 Pacific Blue (103230, BioLegend), CD11c PE-Cy7 (117318, BioLegend), CD80 APC-Fire750 (104738, BioLegend), CD86 APC (7086281, eBioscience), TCRβ chain APC-Cy7 (109219, BioLegend), CD4 PE-Cy7 (100422, BioLegend), CD8a PerCP (100732, BioLegend), PD-1 PE (1299858, eBioscience), Streptavidin-APC (17431782, eBioscience), CD44 FITC (103021, BD Biosciences), CD45 eFluor506 (69045182, eBioscience), IFN-γ APC (505809, BioLegend), IL-10 FITC (505005, BioLegend), IL-17 PE (559302, BD Biosciences), FoxP3 APC (17577382, eBioscience), CD138 PE (5537, BD Biosciences), CD45R/B220 APC-Cy7 (103224, BioLegend), Streptavidin-V450 (560797, BD Biosciences), GL7 FITC (553666, BD Biosciences), Fas PE (554258, BD Biosciences), TCRβ chain PerCP (109227, BioLegend), IgG1 FITC (553443, BD Biosciences), IgG3 FITC (553403, BD Biosciences), BrdU FITC (364103, BioLegend), NK1.1 PE (108707, BioLegend), ICOSL PE (107405, BioLegend), FoxP3 PE (12577382, eBioscience), IL-10R PE (112705, BioLegend), CD11b PE (101207, BioLegend), IFN-γ APC (505809, BioLegend), IgG2a APC (407109, BioLegend), IgG2b APC (406711, BioLegend), Ki67 APC (65405, BioLegend), Annexin V APC (640941, BioLegend), CD8 APC (17008183, eBioscience), CD19 APC (115511, BioLegend), NK1.1 APC-Cy7 (108723, BioLegend), I-A/I-E Pacific Blue (107619, BioLegend), CD138 BV421 (142508, BioLegend), CD4 Pacific Blue (100427, BioLegend), CD11b Biotin (101203, BioLegend), CD11c Biotin (117303, BioLegend), CD4 Biotin (100404, BioLegend), CD8 Biotin (100704, BioLegend), STAT3 Phospho (Tyr705)Alexa Fluor 647 (651008, Biolegend). 

### 4.8. Immune Cell Phenotyping 

The following immune cell subsets were identified in NZB/W F1 animals treated in vivo with α-IL-10R antibodies or isotype control, ex vivo or after in vitro culture using cells of untreated NZB/W F1 mice: B cells (% TCRβ^−^B220^+^/live cells); germinal center B cells (% Fas^hi^GL7^hi^/B cells); plasma cells/-blasts (% CD138^hi^/live cells); IgG1, IgG2a, IgG2b and IgG3 on plasma cells/-blasts; CD4^+^ and CD8^+^ T cells (%TCRβ^+^B220^−^CD4^−^CD8^+^ or TCRβ^+^B220^−^CD8^−^CD4^+^/live cells); expression of ICOS, pSTAT3, IFN-γ, IL-17, IL-10 and CD44^hi^ on CD4^+^ or CD8^+^ T cells; regulatory T cells (T_reg_) (% FoxP3^+^/CD4^+^ T cells); follicular B-helper T cells (T_FH_) (% CXCR5^hi^PD1^hi^/CD4^+^ T cells); DC (CD11c^hi^CD19^−^TCRβ^−^NK1.1^−^/live cells); neutrophils (Ly6G^hi^CD11b^+^CD19^−^TCRβ^−^NK1.1^−^/live cells); CD11b^+^ monocytic cells (Ly6G^−^CD11b^+^CD19^−^TCRβ^−^NK1.1^−^/live cells); and expression of ICOSL, I-A/I-E, CD80 or CD86 on monocytic cells, B cells and DC. In kidney, infiltrating leukocytes were determined as CD45^+^/live cells.

### 4.9. Real-Time Quantitative PCR (RT-PCR)

Total RNA from spleen was extracted using TRIzol reagent (Invitrogen, Thermo Fisher Scientific, Freiburg im Breisgau, Germany), and total RNA from FACS-purified immune cell subsets was extracted using the RNeasy^®^ Micro Kit (Qiagen, Hilden, Germany). The QuantiTect Reverse Transcription Kit (Qiagen) was used for cDNA synthesis according to the manufacturer’s instructions. Transcripts were quantified by real-time quantitative PCR (RT-PCR) on a StepOnePlus™ Real-Time PCR System (Applied Biosystems, Thermo Fisher Scientific, Freiburg im Breisgau, Germany) with pre-designed TaqMan Gene Expression Assays and reagents according to the manufacturer’s instructions (Applied Biosystems, Thermo Fisher Scientific, Freiburg im Breisgau, Germany). Probes with the following Applied Biosystems assay identification numbers were used: Mm99999915_g1 (*GAPDH*), Mm01288386_m1 (*IL-10*), Mm00434151_m1 (*IL-10R*), Mm00517640_m1 (*IL-21*), Mm02581355_s1 (*cMAF*), Mm00461162_m1 (*IL-27*). For each sample, *mRNA* abundance was normalized to the amount of GAPDH and is presented in arbitrary units (A.U.). 

### 4.10. Assessment of Cytokines (ELISA)

Cytokine concentrations in culture supernatants were assessed by ELISA according to standard protocols (IL-6 DuoSet Elisa kit, R&D systems, Wiesbaden-Nordenstadt, Germany, DY401; IL-1β DuoSet Elisa kit, R&D systems, DY406; TNF-α DuoSet Elisa kit, R&D systems, DY410). The absorbance at 450 nm was measured using the Spark^®^ 10 M multimode microplate reader (Tecan, Crailsheim, Germany).

### 4.11. Statistical Analysis

For statistical analysis, Instat software (GraphPad Prism version 9.0.0, San Diego, California, USA) was used. Statistical comparison between two and more than two experimental groups was performed using a Mann–Whitney test (unpaired analysis), Wilcoxon test or one-sample *t* test (paired analysis) and Friedman test (paired analysis), respectively. Outliers were determined by the ROUT method and excluded from statistical analysis and graphic representation. Generally, *p* < 0.05 was considered significant. In all figures, *p* > 0.2 is indicated as ns—not significant.

## Figures and Tables

**Table 1:** In vivo effects of α-IL-10R application on immune status in NZB/W F1 animals.

	Healthy	Sick		
	Untreated	Untreated	α-IL-10R-Treated	
Spleen	Mean (±SEM)	Mean (±SEM)	Mean (±SEM)	*p*-Value
CD11b^+^Ly6G^hi^ neutrophils/live cells (%)	0.1491 (0.05937)	0.3301 (0.07797)	0.9236 (0.1183)	0.0007
CD11c^hi^ DC/live cells (%)	0.04564 (0.00500)	0.2169 (0.02838)	0.2435 (0.04614)	ns
CD80^+^/DC (MFI)		264.0 (30.49)	226.0 (15.13)	ns
CD86^+^/DC (MFI)		1486 (60.27)	1582 (85.68)	ns
I-A/I-E^+^/DC (MFI)		11329 (1088)	9366 (754.8)	0.1500
ICOSL^+^/DC (MFI)		378.1 (28.30)	383.7 (58.30)	ns
CD11b^+^ monocytic cells/live cells (%)	0.4704 (0.1371)	0.8572 (0.1792)	2.034 (0.2418)	0.0010
CD80^+^/MC (MFI)		381.2 (52.22)	370.4 (48.49)	ns
CD86^+^/MC (MFI)		765.1 (20.96)	803.2 (41.81)	ns
I-A/I-E^+^/MC (MFI)		1220 (40.97)	1182 (37.34)	ns
ICOSL^+^/MC (MFI)		528.8 (17.39)	557.6 (35.00)	ns
TCRβ^+^CD4^+^ T cells/live cells (%)	17.57 (0.2906)	16.05 (0.7924)	12.93 (0.9114)	0.0226
CD44^hi^/CD4^+^ T cells (%)	12.42 (1.069)	52.2 (4.080)	61.09 (4.546)	0.1226
CXCR5^hi^PD1^hi^/CD4^+^ T cells (%)	0.3583 (0.02892)	1.802 (0.2429)	3.820 (0.4114)	0.0002
IFN-γ^+^/CD4^+^ T cells (%)	5.845 (0.5039)	13.36 (1.763)	12.95 (1.480)	ns
IL-17^+^/CD4^+^ T cells (%)	0.0798 (0.00634)	0.2590 (0.05745)	0.3790 (0.03174)	0.0973
IL-10^+^/CD4^+^ T cells (%)	1.122 (0.1058)	5.058 (0.6076)	6.345 (0.7977)	0.1674
FoxP3^+^/CD4^+^ T cells (%)	14.58 (0.5437)	2311 (1.654)	20.24 (1.640)	0.1911
Annexin V^+^/CD4^+^ T cells (%)		11.05 (1.202)	20.59 (3.446)	0.0548
BrdU^+^/CD4^+^ T cells (%)		2.164 (0.2925)	3.615 (0.4924)	0.0184
TCRβ^+^CD8^+^ T cells/live cells (%)	11.38 (0.3842)	7.312 (0.9999)	5.196 (0.8768)	0.1339
CD44^hi^/CD8^+^ T cells (%)	8.280 (0.7897)	16.92 (0.7688)	17.70 (1.406)	ns
IFN-γ^+^/CD8^+^ T cells (%)	14.02 (0.7795)	10.16 (1.259)	11.47 (1.047)	ns
Annexin V^+^/CD8^+^ T cells (%)		9.525 (0.8064)	16.09 (2.492)	0.0352
BrdU^+^/CD8^+^ T cells (%)		1.098 (0.2335)	1.180 (0.2155)	ns
TCRβ^−^B220^+^ B cells/live cells (%)	31.10 (1.002)	34.20 (0.8938)	32.55 (1.1419)	ns
Fas^hi^GL7^hi^/B220^+^ B cells (%)	0.3167 (0.03127)	1.181 (0.3272)	2.983 (0.2629)	0.0007
CD80^+^/B220^+^ B cells (MFI)		141.8 (15.79)	120.0 (6.43)	ns
CD86^+^/B220^+^ B cells (MFI)		631.0 (36.82)	750.3 (27.73)	0.0358
I-A/I-E^+/^B220^+^ B cells (MFI)		13046 (665.9)	13279 (397.8)	ns
ICOSL^+^/B220^+^ B cells (MFI)		189.2 (12.67)	169.3 (22.63)	ns
Annexin V^+/^B220^+^ B cells (%)		26.80 (1.905)	28.51 (1.689)	ns
BrdU^+/^B220^+^ B cells (%)		5.336 (0.6873)	8.005 (0.6776)	0.0020
CD138^hi^ plasma cells/live cells (%)	0.8245 (0.1023)	3.359 (0.3818)	5.826 (0.6163)	0.0056
IgG1^+/^CD138^hi^ (%)	8.362 (2.790)	5.365 (0.9021)	10.35 (0.8451)	0.0011
IgG2a^+/^CD138^hi^ (%)	6.1013 (0.7867)	21.88 (2.451)	22.04 (3.053)	ns
IgG2b^+/^CD138^hi^ (%)	8.2868 (0.9861)	7.915 (1.132)	16.39 (0.6913)	<0.0001
IgG3^+/^CD138^hi^ (%)	6.318 (0.8690)	13.58 (1.098)	15.77 (1.274)	0.1914

Broad immune status evaluation in spleens of untreated, 15 week old healthy NZB/W F1 (*n* = 6 mice) and NZB/W F1 animals after 6 weeks of treatment with α-IL-10R or isotype control (*n* (α-IL-10R) = 11 mice; *n* (isotype) = 13 mice) that was initiated at an age of 20–22 weeks in matched animals with detectable α-dsDNA antibodies. Results are expressed as mean ± SEM. The *p* value was calculated using a Mann–Whitney test to determine the difference between isotype- and α-IL-10R-treated animals.

## Data Availability

https://www.mdpi.com/ethics.

## Supplementary Materials

The following Supplementary Materials are available online at https://www.mdpi.com/1422-0067/22/3/1347/s1, Figure S1: IL-10R expression on main immune cell populations in lupus-prone NZB/W F1 mice.

## Abbreviations

α-dsDNA	Anti-double stranded DNA autoantibodies
α-IL-10α-IL-10R	Anti-IL-10 antibodiesAnti-IL-10 receptor antibodies
A.U.	Arbitrary units
BC	B cells
B_reg_	regulatory B cells
BrdU	bromdesoxyuridin
CFDA-SE	carboxyfluorescein diacetate succinimidyl ester
DC	dendritic cells
ELISA	enzyme-linked immunosorbent assay
FACS	fluorescence-activated cell sorting
GC	germinal center
IFN-γ	interferon-γ
IgG	immunoglobulin G
IL-1β	interleukin-1β
IL-6	interleukin-6
IL-10	interleukin-10
IL-10R	interleukin-10 receptor
IL-21	interleukin-21
IL-27	interleukin-27
LPS	lipopolysaccharide
MC	monocytic cells
MFI	mean fluorescence intensity
NZB/W F1	New Zealand black x New Zealand white F1
PC	plasma cells
PMA	phorbol 12-myristate-13-acetate
RT-PCR	real-time quantitative PCR
T_FH_	follicular B-helper T cells
T_h1_	T helper cell 1
T_h17_	T helper cell 17
TLR	toll-like receptor
TNF-α	tumor necrosis factor-α
T_reg_	regulatory T cells
TR1	inducible T regulatory type 1
W	weeks
