# Supplementary material for: Dual Role of Interleukin-10 in Murine NZB/W F1 Lupus
Source: Int J Mol Sci. 2021 Jan 29;22(3):1347. doi: 10.3390/ijms22031347 (PMC7866297; doi:10.3390/ijms22031347)
Supplement: Supplementary file 1 [file ijms-22-01347-s001.pdf]

Supplement Figure 1

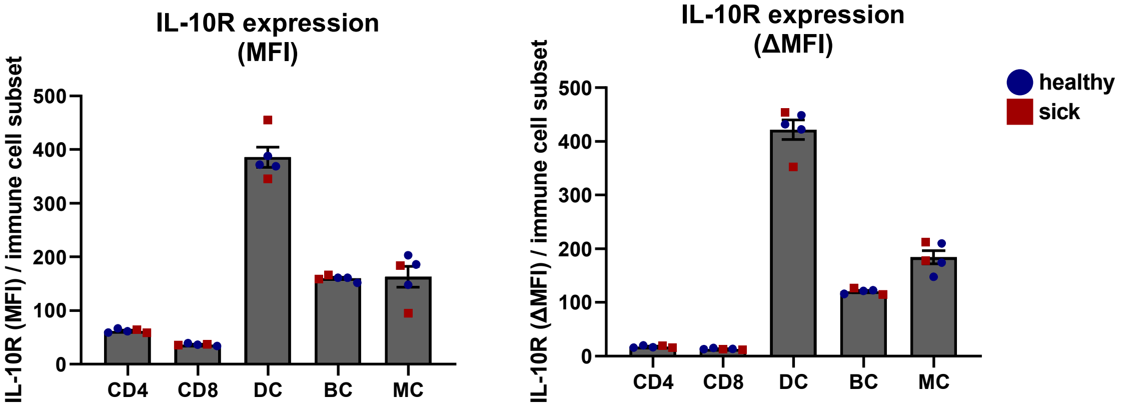

Supplement Figure 1. IL-10R expression on main immune cell populations in lupus-prone NZB/W F1 mice.

Splenocytes from 14 weeks old healthy (n = 3 mice) and 28 weeks old NZB/W F1 animals with established autoantibodies and beginning nephritis (sick; n = 2 mice) were examined by flow cytometry for expression of IL-10R on CD4 and CD8 T cells, dendritic cells (DC), B cells (BC) and monocytic cells (MC). Depicted are MFI (left figure) and ΔMFI (right figure; calculated by subtracting the unstained MFI from the stained MFI).
